# Human Papillomavirus and students in Brazil: an assessment of knowledge of a common infection – preliminary report^☆^

**DOI:** 10.1016/j.bjorl.2016.02.006

**Published:** 2016-04-22

**Authors:** João Cesar Frizzo Burlamaqui, Ana Carolina Cassanti, Gabriela Bastos Borim, Edward Damrose, Luisa Lina Villa, Leonardo Silva

**Affiliations:** a Faculdade de Ciências Médicas da Santa Casa de São Paulo, São Paulo, SP, Brazil; b Stanford University, California, USA; c Universidade de São Paulo (USP), Faculdade de Medicina, Departamento de Radiologia e Oncologia, São Paulo, SP, Brazil

**Keywords:** Brazil, Viruses, Sexual behavior, Policy, Brasil, Vírus, Comportamento sexual, Política

## Abstract

**Introduction:**

Human Papillomavirus (HPV) infection is the most prevalent sexually transmitted disease worldwide. One of the barriers to the implementation of prevention programs against the disease is the limited knowledge possessed by most populations regarding the virus and its possible consequences.

**Objective:**

The purpose of this study was to evaluate the knowledge of Brazilian college students on transmission, clinical manifestations, and diseases correlated with HPV, highlighting the poor knowledge of a very common infection.

**Methods:**

A total of 194 students answered a questionnaire about transmission, clinical features and the possible consequences of persistent HPV infection. The questionnaire was self-applied under the supervision of the authors.

**Results:**

The clinical manifestations of HPV infection were not clear to most students. Incorrect assumptions of the clinical manifestations of HPV infection included: bleeding (25%), pain (37%) and rashes (22%). Twelve per cent of respondents did not recognize warts as an HPV-related disease. Regarding potential consequences of persistent infection, students did not recognize a relationship between HPV and laryngeal carcinoma (80.9%), pharyngeal carcinoma (78.9%), anal carcinoma (73.2%), vulvar carcinoma (65.4%) and vaginal carcinoma (54.6%). Large portions of the population evaluated were unaware of modes of HPV transmission beyond genital contact.

**Conclusion:**

Knowledge of HPV by the population evaluated in this study is partial and fragmented. Lack of knowledge may contribute to the further spread of the disease. Public health policies for education and guidance of the population should be implemented in Brazil.

## Resumo

**Introdução:**

A infecção pelo papilomavírus humano (HPV) é a doença sexualmente transmissível mais prevalente em todo o mundo. Uma das barreiras para a implementação de programas de prevenção contra a doença é o conhecimento limitado da maioria das populações sobre o vírus e suas possíveis consequências.

**Objetivo:**

O objetivo deste estudo foi avaliar o conhecimento dos estudantes universitários brasileiros sobre a transmissão, manifestações clínicas e doenças correlacionadas com o HPV, com destaque para o pouco conhecimento de uma infecção muito comum.

**Método:**

Um total de 194 estudantes respondeu a um questionário sobre a transmissão, características clínicas e as possíveis consequências da infecção persistente pelo HPV. O questionário foi autoaplicado, sob a supervisão dos autores.

**Resultados:**

As manifestações clínicas da infecção pelo HPV não eram claras para a maioria dos estudantes. As suposições incorretas das manifestações clínicas da infecção pelo HPV incluíam: hemorragia (25%), dor (37%) e erupções cutâneas (22%) – 12% dos entrevistados não reconheciam as verrugas como uma doença relacionada ao HPV. Quanto às potenciais consequências da infecção persistente, os alunos não reconheciam uma relação entre HPV e câncer de laringe (80,9%), carcinoma da faringe (78,9%), carcinoma anal (73,2%), carcinoma vulvar (65,4%) e carcinoma vaginal (54,6%). Grandes porções da população avaliada desconheciam os modos de transmissão do HPV além do contato genital.

**Conclusão:**

O conhecimento de HPV pela população avaliada neste estudo é parcial e fragmentado. A falta de conhecimento pode contribuir para a propagação da doença. Políticas públicas de saúde para a educação e orientação da população devem ser implementadas no Brasil.

## Introduction

Human Papillomavirus (HPV) is the etiological agent of the most common viral infection of the genital tract worldwide. The virus is also related to a wide range of disorders in children and adults of both genders. The clinical impact of HPV-related disease encompasses a range of benign and malignant disorders.^1^

HPV infection is the most prevalent sexually transmitted disease worldwide, with over 14 million new cases reported in the United States in 2008. Taking into account all infected individuals, it is estimated that about 79 million Americans were infected with HPV in 2008.^2^

The human papillomavirus is a DNA virus of the *Papillomaviridae* family. HPV infects skin and mucosa. Based on differences in the genomic sequence of L1, the gene encoding the major capsid protein, more than 190 types of HPV have been identified by molecular analysis.^3^

HPV is classified into high- and low-risk types according to their potential to induce cancer in infected tissues.^1^ Although sexual contact is the most widely accepted mode of transmission, other forms of contamination are described in the literature.^4^ Vertical transmission (infection of infants by passage through a contaminated birth canal) is 231 times higher in women with vaginal/vulvar condyloma than in women without clinically evident disease. Transmission of HPV through subclinical infection may also be an important mode of infection, perhaps the highest risk factor when studying maternal-fetal transmission rate.^5^

The clinical manifestations of HPV-related diseases vary depending upon the HPV type and the site of inoculation, but the wart is considered the classic primary lesion of infection.^6^

While persistent infection in the cervix by high-risk HPV (16 and 18) has long been considered the causative factor in cervical and uterine cancer, it has been only recently recognized that this same process accounts for cancer of the anus, penis, vagina, vulva and oropharynx.^7^, ^8^

HPV infection with serotypes 6 and 11 (low-risk) causes diseases such as recurrent laryngeal and oral papillomatosis.^9^

Considering the high prevalence of HPV-associated disease and its potential for recurrence and malignant conversion, costs related to diagnosis and treatment have a major impact on health systems worldwide. For example, in 2003 alone the United States spent $418 million dollars for the treatment of HPV-related diseases, excluding the amounts spent on cervical disease.^10^

In 2012, the Italian health system spent 528.6 million Euros for the treatment of HPV-related diseases (cervical cancer, vulvar cancer, vagina, anus, penis, head and neck, genital warts and respiratory papillomatosis).^11^

In addition to the extremely high costs for health systems worldwide, HPV-related diseases are associated with high rates of morbidity and mortality.^12^

As with other sexually transmitted diseases, prevention remains the best option for controlling HPV-related diseases, particularly since there is no curative treatment.^9^ Among the different forms of prevention available for HPV-related diseases, vaccination has demonstrated cost-effectiveness in several programs around the world.^9^

However, one of the barriers to the implementation of these programs is the limited knowledge possessed by most populations^13^, ^14^, ^15^, ^16^ regarding the virus and its possible consequences.

Studies in different parts of the world have identified that there is not a proper understanding of the topic when considering sexually active young people.^17^, ^18^, ^19^

Positively, countries that have implemented immunization programs have observed that vaccination campaigns have contributed to increasing levels of awareness of the issue. These campaigns, however, have been primarily focused on cervical cancer, while the information provided about other HPV-related diseases is limited.^20^

Although the scientific literature provides strong evidence about the relationship between infection by high-risk HPV and the development of oropharyngeal cancer popular knowledge regarding this tumor is inadequate or inexistent.^21^

In a study of 2.126 American adults evaluating overall knowledge of head and neck cancers, less than 1% of participants recognized HPV infection as a possible risk factor for cancer of the mouth and pharynx.^22^

Interestingly, there is a 10-fold difference in the rates of oral infection (4%) and genital infection (40%) in men. Despite relatively low infection rates, development of benign and malignant lesions in the head and neck is increasing at an alarming rate.^23^

Sexual behavior has been related to the increasing rate of oral HPV infection, but there is no consensus in the literature on the subject. Risk factors may include the number of lifetime sexual partners and the increasing practice of oral sex.^24^, ^25^

Subclinical infection may facilitate dissemination of disease to uninfected partners. Condom use should always be encouraged but may not provide total protection against HPV infection because the virus may be shed from sites not protected by the condom.^26^

The purpose of this study was to evaluate the level of knowledge of college students on issues involved in transmission of the virus, clinical manifestations and potential consequences of persistent HPV infections, identifying gaps in knowledge and thus contributing to educational campaigns.

## Methods

This cross-sectional study was approved by the Ethics and Research Committee (CAAE: 20424413.4.0000.5479). Three hundred students of the first two years of the schools of Medicine, Nursing and Speech-Language Pathology and Audiology were invited to participate in this study. The questionnaire was self-applied and the subjects were organized into groups in order to minimize interference with the curriculum of each of the colleges. The authors supervised the completion of the questionnaires. Each questionnaire was identified only by number for the purpose of maintaining the anonymity of each participant.

## Results

Of the 300 students invited to participate in the study, 194 agreed to participate and fully completed the questionnaires. No questionnaire was excluded.

Most of the subjects (92.8%) correctly indicated that the greater the number of sexual partners the higher the risk of HPV infection. Results regarding transmission are displayed in Table 1. Most respondents recognized genital to genital contact as a common mode of transmission, but knowledge regarding other modes of transmission was limited. Most respondents underestimated the risk of maternal-fetal transmission.

**Table 1:** Answers about the modes of transmission of HPV.

Transmission	Yes *n* (%)	No *n* (%)
Genital	191 (98.5)	3 (1.5)
Orogenital	127 (65.5)	67 (34.5)
Anogenital	125 (54.1)	89 (45.9)
Saliva	19 (9.8)	175 (90)
Transplacental	37 (19.1)	157 (80.9)
Vaginal delivery	75 (38.7)	119 (61.3)
Cesarean section	16 (8.2)	178 (91.8)

Regarding protective measures, 51% of participants stated that condoms provide total protection against HPV infection during sexual intercourse, 12.4% could not answer this question and 36.6% reported that such protection would not be fully effective. When asked if they could infect their partners even when asymptomatic 88.7% of respondents answered positively, 2.2% negatively and 8.8% did not know.

Regarding development of malignancy, the risk of cervical and penile cancer was most readily recognized, with much lower appreciation for cancer at other sites (Table 2). Uncertainty regarding cancer risk in these other sites was also evident.

**Table 2:** Proportion of responses about the participation of HPV in the pathogenesis of cancer in different anatomical sites.

Neoplasia	Yes (%)	No (%)	Don’t know (%)
Pharynx	41 (21.1)	68 (35.1)	85 (43.8)
Larynx	37 (19.1)	66 (34)	91 (46.9)
Vulva	67 (34.5)	41(21.1)	86 (44.3)
Vagina	88 (45.4)	34 (17.5)	72 (37.1)
Penis	184 (94.8)	4 (2.1)	6 (3.1)
Anus	52 (26.8)	51 (26.3)	91 (46.9)
Cervical	177 (91.2%)	11(5.7%)	6 (3.1%)

**Figure 1:**
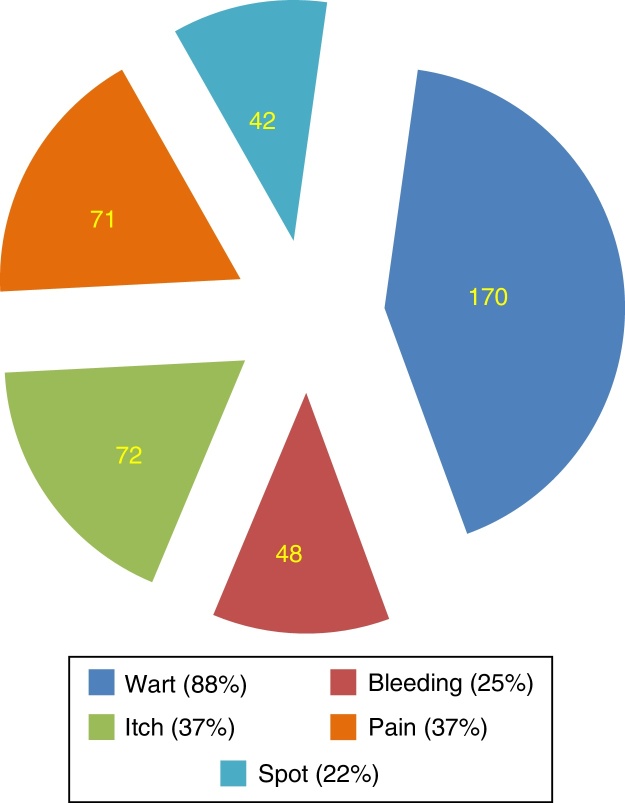
Signs and symptoms of HPV infection in accordance with the understanding of the surveyed participants.

Regarding signs and symptoms of HPV infection, participants reported warts to be the main symptom (88%). Symptoms such as bleeding (25%), itching (37%), and spot (22%) were incorrectly identified as part of routine symptomatology (Fig. 1).

## Discussion

According to the United States Centers for Disease Control, 49% of HPV infections occur in people between 15 and 24 years of age, with significant treatment-related costs.^27^

Low socioeconomic and educational status is correlated with a poor prognosis in HPV-related diseases, such as Recurrent Respiratory Papillomatosis, as well as with low rates of adherence to infection prevention programs.^28^

In Brazil, 44.9% of the population over age 15 has either no education or incomplete elementary school education, while 19% have incomplete middle school education.^29^

It is important to consider that although the educational level of the study group was higher than that of the general population, the participants had no formal education in HPV or its related diseases. Thus, the knowledge of this group is representative of their peers. Our results show good knowledge of respondents on some issues, but in other important areas this knowledge is partial and fragmented.

The vast majority of the subjects in the study (92.8%) indicated that the greater the number of sexual partners, the greater the risk of HPV infection. This result was also observed in other studies.^25^, ^30^ Genital to genital transmission was readily recognized as a significant factor for HPV infection. Unfortunately, alternative forms of transmission such as orogenital and anogenital were recognized by less than half of the respondents, an important deficit in understanding what are considered to be safe sexual practices. This data is particularly troubling when considering the possible consequences of persistent infections in these sites (malignant transformation) and deserves special attention for the formulation of future educational health policies.^24^, ^30^

The subjects in this work belong to an age with a high number of sexual partners and low frequency of condom use.^31^

Our results indicate that the majority (63.4%) of respondents feel fully protected against HPV infection when using condoms. Alternative clinical presentations of viral infection (RRP and cancer) were not recognized by the assessed population, a fact that should also be considered in prevention programs.

The vast majority of respondents did not recognize the possibility of transplacental transmission of the virus or even if transmission can occur during vaginal childbirth. When we consider the age and years of reproductive life of the studied population, inadequate understanding of HPV in this group represents a serious and dangerous knowledge gap.

With respect to HPV-related disease, cervical cancer was most readily recognized by study participants (91.23%). Unfortunately, there was poor recognition of the association of infection with other cancers. These data are consistent with the epidemiological data that has been presented in recent years in relation to such diseases.^25^, ^32^

While educational efforts have been successful in establishing recognition of the relationship between HPV and cervical cancer, more aggressive efforts must be made to firmly establish this same recognition with other cancers. Our data indicate that 88.7% of respondents readily recognized the potential for spreading the disease when asymptomatic. Most respondents recognized the wart as the classic symptom of infection, a rate greater than that obtained by other authors,^33^ but they incorrectly identified other signs and symptoms as components of active infection (Fig. 1). Self-examination may be critical to recognition of active infection, and should be encouraged. Early detection of active infection is important as late diagnosis is associated with higher complication rates.^23^, ^34^

However, the inability to correctly identify signs of active infection, as demonstrated in this study, represents a critical barrier that must be overcome for the successful implementation of a self-examination program.

Taking into account the potential severity and costs related to HPV-associated diseases, the role of prevention is unquestionable. However, for individual protective measures to be effective it is necessary that a minimum level of knowledge on the subject is achieved by the population.

From the results obtained in this work we have identified gaps in knowledge about HPV infection. The data in this study was obtained from a group representing above-average education level in Brazil, highlighting the deficiencies in current educational programs in this country regarding HPV infection. It is essential that the Brazilian public policy regarding this subject be reevaluated.

However, it has been observed by other authors that an increase in knowledge of the problem is not reflected in awareness of the importance of the preventive measures to be adopted. These authors recommended that information should be direct and accompanied by arguments that cannot be misinterpreted.^35^

The facts that our sample was not representative of the entire Brazilian population as well as the limiting of recruitment to only one university are potential limitations of this study. Nevertheless, we believe that this work can provide useful information for policy guidance for the population.

Knowledge gaps about HPV infection and its potential consequences can decrease the efficiency and scope of prevention programs. Although vaccination programs are already in place for a large portion of the population they should be supplemented with information for the entire population. Otherwise, the efficacy of vaccination programs may be compromised. It is essential to promote educational campaigns about the risks of transmission, forms of protection and consequences of HPV-related diseases in order to minimize the individual morbidity and to minimize costs to the health system. Public health policies for education and guidance of the population should be implemented in Brazil.

## Conclusion

Knowledge of HPV by the population evaluated in this study is partial and fragmented. Lack of knowledge may contribute to the further spread of the disease. Public health policies for education and guidance of the population should be implemented in Brazil.

## Conflicts of interest

The authors declare no conflicts of interest.
